# Cerebral Venous Thrombosis in a Patient with Clinically Isolated Spinal Cord Syndrome

**DOI:** 10.1155/2013/364869

**Published:** 2013-08-05

**Authors:** Jasem Yousef Al-Hashel, Samar Farouk Ahmed, K. J. Alexnader, Walaa Ahmed

**Affiliations:** ^1^Neurology Department, Ibn Sina Hospital, P.O. Box 25427, 13115 Safat, Kuwait; ^2^Kuwait University, P.O. Box 24923, 13110 Safat, Kuwait; ^3^Minia University, P.O. Box 61519, Minia City, Minia 61111, Egypt

## Abstract

*Background*. The association between cerebral venous thrombosis (CVT) and multiple sclerosis (MS) has already been reported in patients with clinically definite MS in relation to intravenous methylprednisolone (IVMP) or previously performed lumbar puncture (LP). *Case Summery*. We report a 29-year-old Indian female who presented with a clinically isolated spinal cord syndrome according to the revised 2010 McDonald Criteria. She developed CVT after a lumbar puncture and two days of finishing the course of IVMP. *Conclusion*. We conclude that the sequence of doing lumbar puncture followed by high-dose IVMP may increase the risk of CVT. A prophylactic anticoagulation may be indicated in this setting.

## 1. Introduction

Cerebral venous thrombosis (CVT) may occur at any age and may be idiopathic or secondary to various causes. Several cases of CVT have been described in patients with multiple sclerosis (MS) [1]. In the majority of these cases, lumbar puncture followed by IVMP was suspected as the cause of CVT [2]. MS may present with a wide range of central and even peripheral nervous system symptoms and signs, so the coincidence of other neurological disorders in MS patients may remain undetected. Therefore, more clinical evaluations and investigations should be considered in any MS patient who presents with new atypical symptoms [1].

We report a case with multifocal clinically isolated syndrome (CIS). This patient developed CVT following lumbar puncture and 2 days after IVMP pulse therapy.

## 2. Case Presentation

A 29-year-old Indian female, who was two months postpartum, presented to our hospital with history of numbness of her lower limbs that ascended up to her chest. The numbness began about ten months back and remained stationary during the pregnancy. She did not seek any medical advice at that time. Two months postpartum, the numbness became more severe and annoying. She did not have any other neurological symptoms. There was no history of any oro-genital ulcers, redness of eyes, or abortions. She denied any previous similar attacks. She had skin lesions over the elbow and knee joints suggestive of psoriasis, which was treated with topical corticosteroid. On examination she was fully conscious, alert, oriented, and with normal cranial nerve functions. Motor system examination revealed normal muscle bulk, tone, power, and deep tendon reflexes in the upper limbs. Lower limb examination showed normal muscle bulk with hypertonicity and distal weakness of Medical Research Council Power Scale grade 4/5 bilaterally. The deep tendon reflexes were exaggerated in the lower limbs with bilateral clonus and positive Babinski's sign. There was a sensory level at T6 on the left side. 

**Figure 1:**
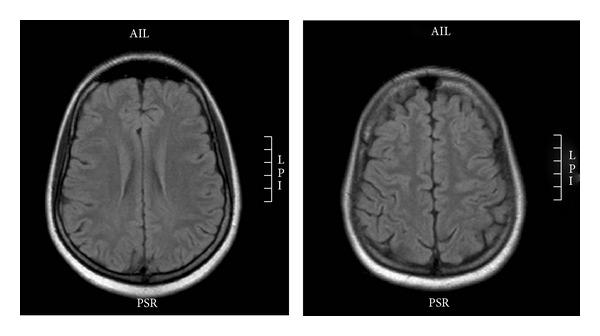
MRI brain axial flair.

**Figure 2:**
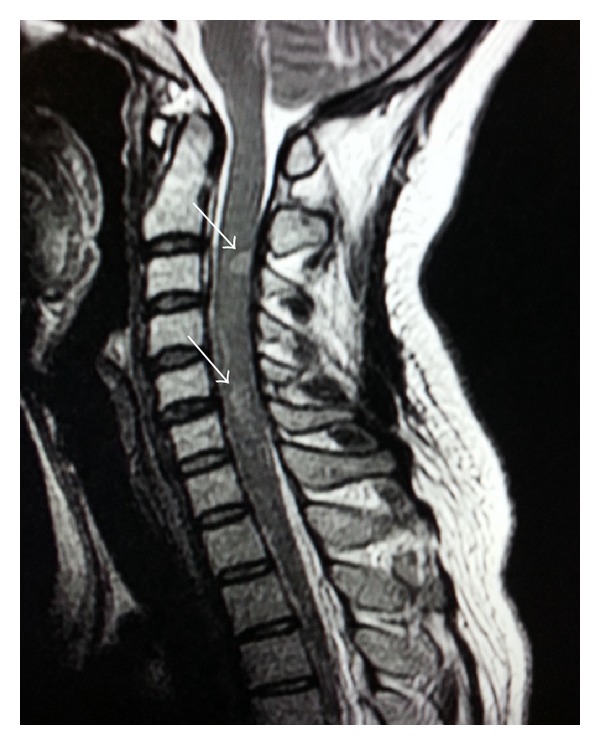
MRI cervicodorsal spine showing multiple plaques of abnormal signal on T2W image (arrows).

Routine blood investigations were normal. Autoimmune profile, vasculitis workup, serum vitamin B12, folate, and thyroid function tests all were normal. Neuromyelitis optica antibody (NMO antibody) was negative. Visual evoked potential (VEP) latency was prolonged bilaterally. Brainstem auditory evoked potential (BAER) and somatosensory evoked potential (SSEP) were normal. A lumbar puncture (LP) study showed CSF with no cells, normal glucose, but elevated protein (640 mg/L). Glucose and cells were normal. CSF oligoclonal band was positive. Brain magnetic resonance imaging (MRI) was normal (Figure 1). Spinal MRI showed multiple hyperintense lesions on T2WI at C2-C3, C3-C4, C5-6, T3-T4, and T5 levels (Figure 2). None of these lesions showed contrast enhancement. 

The patient was diagnosed as a case of multifocal clinically isolated spinal cord syndrome according to the revised 2010 McDonald Criteria [3]. The diagnosis was based on the clinical presentation of monophasic myelitis, abnormal MRI cervicodorsal spine, and abnormal VEP. A course of intravenous methylprednisolone (IVMP) pulse (1 gram daily for 5 days) was started accordingly. 

Two days after the pulse therapy, the patient developed severe headache, right focal with secondary generalized tonic clonic seizures (GTCS), and mild right-sided weakness. The neurological examination showed mild receptive aphasia, right homonymous hemianopia, right-sided hemiparesis, and hemi hypoesthesia including the face.

**Figure 3:**
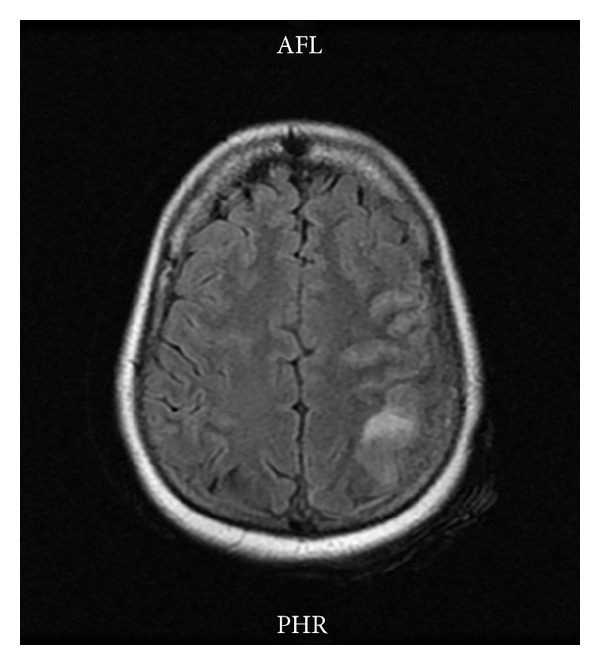
MRI brain T2W axial image showing hyperintense left parietal lesion.

**Figure 4:**
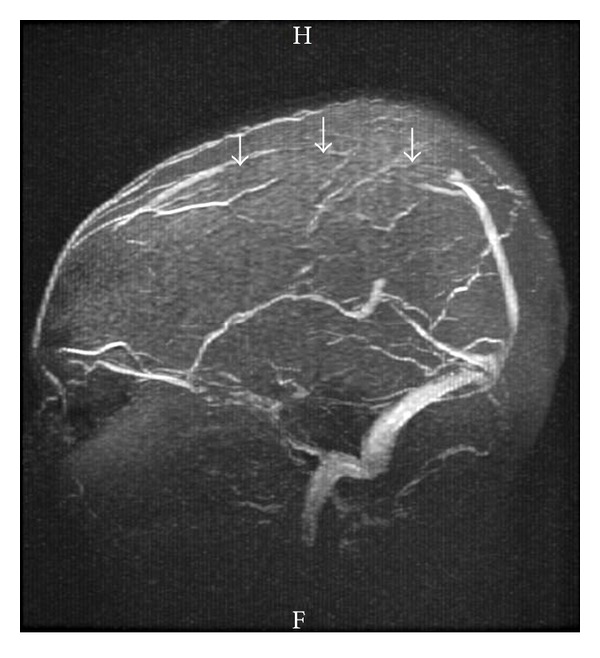
MRV  showing absent flow in the anterior and middle parts (arrows) of the superior sagittal sinus suggestive of venous sinus thrombosis.

 An urgent MRI with MRV was done which showed left parietal hyperintense lesion on T2WI and FLAIR images (Figure 3). The MRV showed filling defect of the superior sagittal sinus thus confirming cerebral venous sinus thrombosis (Figure 4). Thrombophilia workup showed normal levels of protein S, protein C, antithrombin III, Factor V Leiden, serum homocystein and fibrinogen.

She was treated with heparin anticoagulation and was later switched to warfarin. She was given anticonvulsants also and there was no recurrence of any seizure.

## 3. Discussion

Cerebral venous thrombosis is one of the disorders that may complicate the course of disease in MS patients. It should always be suspected in MS patients when postural postlumbar puncture headache evolves into a severe continuous headache with new focal signs, especially hemiparesis or seizure episodes [1]. Our patient, a 29-year-old female with psoriasis and multifocal clinically isolated spinal cord syndrome, had a diagnostic lumbar puncture developed CVT two days after receiving IVMP. All the usual causes of cerebral venous thrombosis were excluded. 

Previous reports described similar cases and they suggested a relationship between the lumbar puncture and the use of corticosteroids to CVT [2, 4, 5]. Städler et al. reported two similar cases [6]. In one case, the course was favorable but the second patient died in spite of intracerebral thrombolysis. 

The cause of CVT is multifactorial, and in less than twenty percent of cases no clear risk factor can be identified [7]. Drugs may increase the risk of CVT. One of the medications which have been under suspicion in this respect is high-dose IVMP used in treating MS patients in acute relapse [8]. A causative link also has been proposed between lumbar puncture and CVT but it was not always the case [9]. In most instances, lumbar puncture played its role whenever another predisposing factor such as high-dose steroid use is in action [2].

The coincidence of CVT and MS has already been described in the literature [4, 9]. Associated thrombophilia is the cause of thrombosis in most of these cases [1]. The high-dose of intravenous methylprednisolone might cause a hypercoagulable state resulting in CVT [10]. Glucocorticoids are commonly thought to be procoagulant, therefore increasing the risk of thromboembolic complications like deep venous thrombosis and pulmonary embolism [11]. In Cushing's syndrome, the hypercoagulable state is due to an increase in plasma levels of clotting factors such as factor VIII complex. A high level of fast-acting plasminogen activator inhibitor and decreased levels of tissue-type plasminogen activator might give impaired fibrinolytic capacity [12]. However, hypercoagulopathy induced by high-dose IVMP is a controversial issue [1]. The study of Frank et al. investigated the effect of high-dose IVMP on sensitive markers of coagulation and fibrinolysis activation. They did not find any evidence for a pronounced acute prothrombotic state induced by this medication [13]. 

Another study of Kalanie et al. [8] tried to examine the possible role of high-dose IVMP in the genesis of venous thrombosis (VT) during treatment of acute relapses in MS. They found that most of their patients who developed CVT were females, as in our case which may denote the role of gender as a predisposing factor for VT. In fact, estrogens have many different effects on the coagulation system, such as increase in the level of factors VII, X, XII, and XIII and reductions in the anticoagulant factors protein S and antithrombin [14].

Previous reports suggested that MS per se may provide a suitable background for VT [9, 15, 16]. It has been suggested that the inflammatory infiltration in MS plaques located close to small- or medium-sized veins could have a role as well [1]. 

Lumbar puncture can be associated with CVT; indeed, some of the postlumbar puncture headaches could be due to CVT [17]. This is explained by low CSF pressure after LP that causes a downward shift of the brain, with traction on the cortical veins and sinuses that may trigger a venous vasodilatation with resultant stasis [10, 18]. Deformation of the venous walls by any mechanism such as rostro-caudal sagging effect with traumatic damage to the fragile venous endothelial wall after LP may induce thrombosis [19].

The presence of psoriasis in our case could increase the risk of CVT as reported in a previous study that psoriasis is associated with a greater risk of incident venous thromboembolism [20]. It could also be comorbid with MS as both are autoimmune T cell-mediated disorders [21, 22].

## 4. Conclusions

Lumbar puncture followed by high-dose steroid treatment for MS relapse may increase the risk of CVT. Hence prophylactic anticoagulation may be indicated in such patients. CVT should be suspected when new symptoms and signs appear in such patients and immediate imaging study including venography will help early diagnosis and treatment of this potentially life-threatening complication.
